# (±)-(4b*S*,8a*R*,10a*S*)-10a-Ethynyl-4b,8,8-trimethyl-3,7-dioxo-3,4b,7,8,8a,9,10,10a-octa­hydro­phenanthrene-2,6-dicarbonitrile

**DOI:** 10.1107/S1600536812041244

**Published:** 2012-10-10

**Authors:** Suqing Zheng, Daniel Resch, Tadashi Honda, Jerry P. Jasinski

**Affiliations:** a Institute of Chemical Biology and Drug Discovery, Stony Brook University, Stony Brook, NY 11794, USA; b Department of Chemistry, Stony Brook University, Stony Brook, NY 11794, USA; c Department of Chemistry, Keene State College, 229 Main Street, Keene, NH 03435-2001, USA

## Abstract

The anti-inflammatory and cytoprotective tricyclic title compound, C_21_H_18_N_2_O_2_, also known as TBE-31, crystallizes with two nearly superimposable mol­ecules in the asymmetric unit. In both mol­ecules, the three ring systems conform to an envelope–chair–planar arrangement. The central ring, in a cyclohexane chair conformation, contains an axial ethynyl group that bends slightly off from a nearby axial methyl group because of the 1,3-diaxial repulsion between the two groups. In the crystal, weak C—H⋯N and C—H⋯O inter­actions form chains along [001].

## Related literature

For anti-inflammatory, growth suppressive, and proapoptotic properties of TBE-31 and the structural assignment of racemic TBE-31 by NMR spectroscopy, see: Honda *et al.* (2007 ▶, 2011 ▶). For inducing NQO1 and GST in the liver, skin, and stomach in mice, see: Dinkova-Kostova *et al.* (2010 ▶). For TBE-31 activity against aflatoxin-induced liver cancer in rats, see: Liby *et al.* (2008 ▶). For reactivity of the non-enolizable cyano­enone in ring *C* of TBE-31 compared to that of MCE-1, see: Dinkova-Kostova *et al.* (2010 ▶). For the biological potency in bioassays for inhibition of inflammation and carcinogenesis and related biological potency, see: Zheng *et al.* (2012 ▶). For the synthesis of TBE-31, see: Honda *et al.* (2011 ▶). For literature on the number of chemical formula units per asymmetric unit, *Z*′, see: Steiner (2000 ▶); Steed (2003 ▶); Gavezzotti (2008 ▶). For ring-puckering parameters, see: Cremer & Pople (1975 ▶). For all-*trans*-perhydro­phenanthrene comparisons, see: Marcos *et al.* (2005 ▶). For a related structure, see: Bore *et al.* (2002 ▶).
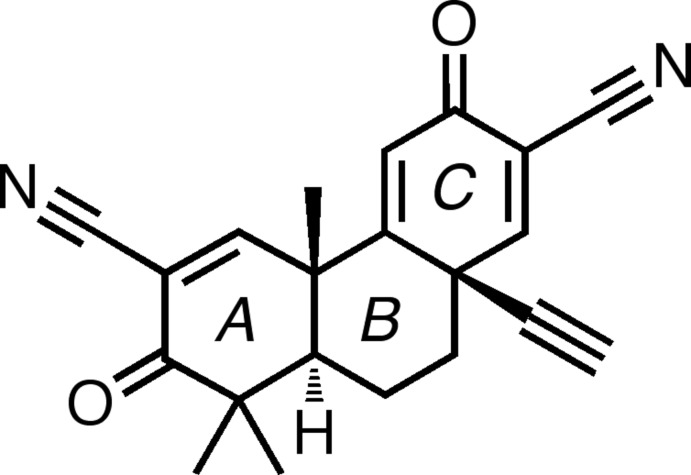


## Experimental

### 

#### Crystal data

C_21_H_18_N_2_O_2_
*M* *_r_* = 330.37
Triclinic, 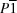

*a* = 7.3012 (2) Å
*b* = 12.9843 (3) Å
*c* = 18.4254 (4) Å
α = 95.051 (2)°
β = 96.284 (2)°
γ = 92.338 (2)°
*V* = 1727.26 (7) Å^3^
*Z* = 4
Cu *K*α radiation
μ = 0.66 mm^−1^
*T* = 299 K
0.71 × 0.46 × 0.29 mm

#### Data collection

Oxford Diffraction Xcalibur Atlas Gemini diffractometer
Absorption correction: Gaussian (*CrysAlis RED*; Oxford Diffraction, 2010 ▶) *T*_min_ = 0.745, *T*_max_ = 0.897
33944 measured reflections
6478 independent reflections
5160 reflections with *I* > 2σ(*I*)
*R*_int_ = 0.035

#### Refinement

*R*[*F* ^2^ > 2σ(*F* ^2^)] = 0.050
*wR*(*F* ^2^) = 0.140
*S* = 1.03
6478 reflections
458 parameters
H-atom parameters constrained
Δρ_max_ = 0.22 e Å^−3^
Δρ_min_ = −0.18 e Å^−3^

### 

Data collection: *CrysAlis PRO* (Oxford Diffraction, 2010 ▶); cell refinement: *CrysAlis RED* (Oxford Diffraction, 2010 ▶); data reduction: *CrysAlis RED*; program(s) used to solve structure: *SHELXS97* (Sheldrick, 2008 ▶); program(s) used to refine structure: *SHELXL97* (Sheldrick, 2008 ▶); molecular graphics: *WinGX* (Farrugia, 1999 ▶); software used to prepare material for publication: *Mercury* (Macrae *et al.*, 2006 ▶), *PLATON* (Spek, 2009) ▶ and *publCIF* (Westrip, 2010 ▶).

## Supplementary Material

Crystal structure: contains datablock(s) I. DOI: 10.1107/S1600536812041244/qk2041sup1.cif

Structure factors: contains datablock(s) I. DOI: 10.1107/S1600536812041244/qk2041Isup2.hkl

Supplementary material file. DOI: 10.1107/S1600536812041244/qk2041Isup3.cml

Additional supplementary materials:  crystallographic information; 3D view; checkCIF report

## Figures and Tables

**Table 1:** Hydrogen-bond geometry (Å, °)

*D*—H⋯*A*	*D*—H	H⋯*A*	*D*⋯*A*	*D*—H⋯*A*
C4*A*—H4*A*⋯N2*B* ^i^	0.93	2.66	3.572 (3)	166
C4*B*—H4*B*⋯N2*A* ^i^	0.93	2.69	3.580 (2)	161
C7*B*—H7*B*⋯O1*B* ^ii^	0.93	2.43	3.246 (2)	146
C13*B*—H13*D*⋯O1*B* ^iii^	0.97	2.38	3.324 (2)	163
C13*B*—H13*C*⋯O2*A* ^iv^	0.97	2.57	3.435 (2)	148
C13*A*—H13*A*⋯O1*A* ^i^	0.97	2.37	3.295 (2)	159

Symmetry codes: (i) 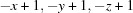; (ii) 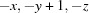; (iii) 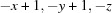; (iv) 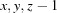.

**Fig. 1.**
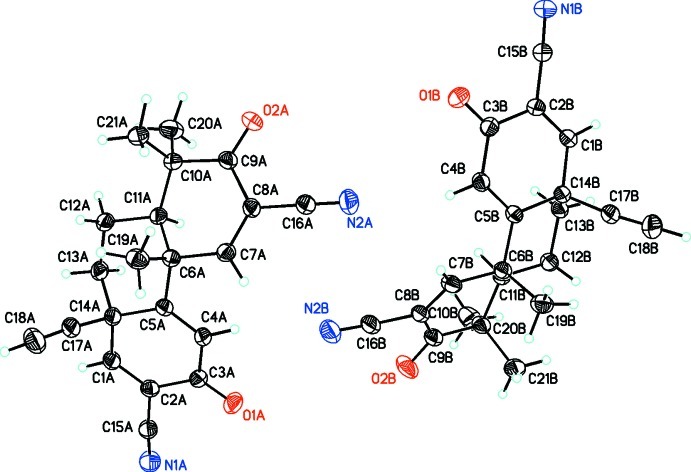
The molecular structure of the title compound with the atom numbering scheme. Displacement ellipsoids are drawn at the 30% probability level. H atoms are presented as small spheres of arbitrary radius.

**Fig. 2.**
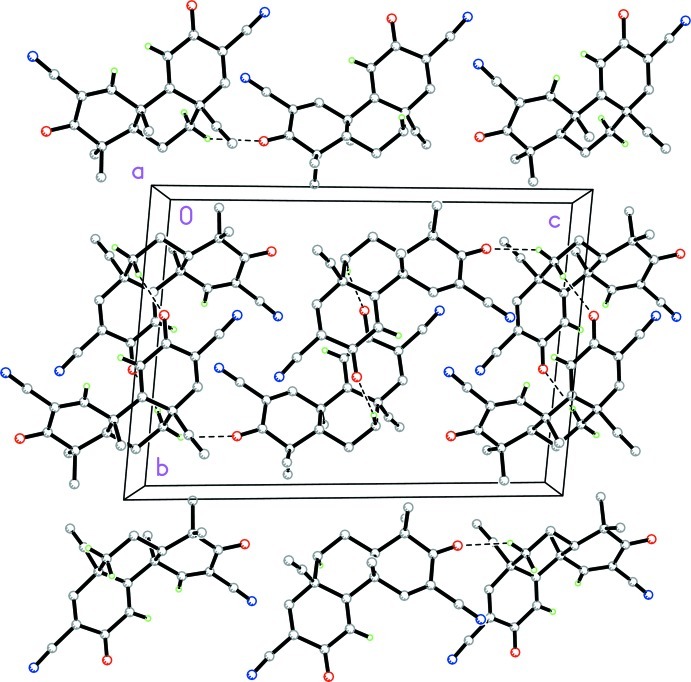
The packing arrangement of the title compound, C21H18N2O2, containing both entaniomers viewed along the a axis. Dashed lines indicate C—H···N hydrogen bonds and weak C—H···O intermolecular interactions forming chains along [001].

**Fig. 3.**
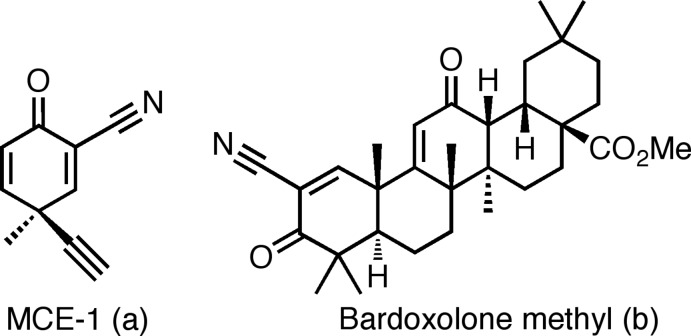
Molecular structure diagram of MCE-1 (a) and bardoxolone methyl (b).

## supplementary crystallographic information

### Comment

The tricyclic compound (±)-(4b*S*,8a*R*,10a*S*)-10a-ethynyl-4b,8,8-trimethyl-3,7-dioxo-3,4b,7,8,8a,9,10,10a-octahydrophenanthrene-2,6-dicarbonitrile (TBE-31) with nonenolizable cyanoenones in rings A and C is a potential anti-inflammatory, growth suppressive, and proapoptotic compound. TBE-31 inhibits nitric oxide (NO) production at low nanomolar concentrations in RAW 264.1 cells and mouse primary macrophages stimulated with IFN-γ (Honda *et al.*, 2007; 2011). TBE-31 induces cytoprotective enzymes HO-1 in RAW cells and in mice (Honda *et al.*, 2007) and NQO1 in Hepa1c1c murine hepatoma cells (Honda *et al.*, 2011). Incorporation of small quantities of TBE-31 in the diet robustly induces NQO1 and GST in the liver, skin, and stomach in mice (Dinkova-Kostova *et al.*, 2010). TBE-31 is orally highly active against aflatoxin–induced liver cancer in rats (Liby *et al.*, 2008). The nonenolizable cyanoenone in ring C of TBE-31 is a highly reactive Michael acceptor and the reactivity is higher than that of MCE-1 (Fig. 3a), which has the same structure as that of ring C (Dinkova-Kostova *et al.*, 2010). Moreover, in this series of Michael acceptors, the reactivity is closely related to the biological potency in the bioassays for inhibition of inflammation and carcinogenesis (Zheng *et al.*, 2012). It has been speculated that the reactivity of the cyanoenone in ring C of TBE-31 would be enhanced because the same structure as that of MCE-1 exists in an unusually unsaturated tricyclic ring system containing eight *sp*^2^ carbons. Thus, we have synthesized TBE-31 from cyclohexanone in 14 steps (Honda *et al.*, 2011) and determined the crystal structure of TBE-31. We herein report the crystal structure determination of the title compound, C_21_H_18_N_2_O_2_, TBE-31, and subsequently discuss its reactivity.

This reported crystal structure confirms previous assignments made by NMR spectroscopy (Honda *et al.*, 2007; 2011). Aside from structural confirmation, the X-ray crystal structure of TBE-31 is interesting due to the presence of two independent molecules in the asymmetric unit (*Z*' = 2) (Fig. 1). Steiner (2000) reported finding 73% of the organic crystal structures in the Cambridge Structural Database (CSD) with a *Z*' = 1 while only 9% have *Z*' = 2. Strong intermolecular interactions have been suspect in the phenomenon of *Z*' > 1, but Steed concluded that it is not possible to use this information to predict the contents of the asymmetric unit (Steed, 2003). Further analysis of the organic crystal structures in the CSD by Gavezzotti showed that certain space groups show higher frequency of *Z*' = 2 (Gavezzotti, 2008). In the space group *P*1, 25% of all the organic crystal structures analyzed by Gavezzotti have *Z*' = 2 (Gavezzotti, 2008). He also found that 64% of all the organic crystal structures with *Z*' = 2 contain the ketone functional group. In addition, hydrogen bond accepting nitrogen groups were found in 58% of the *Z*' = 2 crystal structures (Gavezzotti, 2008). TBE-31 solves in the *P*1 space group, possesses the ketone functional group, and displays weak C—H···N and C—H···O intermolecular interactions (Table 1) forming chains along (001) (Fig. 2). No H-bond donors are present in TBE-31, but it is crystallized from H-bond donating methanol. The structural attributes of TBE-31 imply it is reasonable to have *Z*' = 2. The two molecules in the asymmetric unit (A and B molecule; Fig. 1) are also superimposable (r.m.s.d. = 0.091 (1)Å) by local symmetry.

The crystal structure of the title compound, TBE-31, is the latest addition to an important class of cyanoenone-based drugs. The only previously reported crystal structure of a cyanoenone-based drug, to our knowledge, is methyl 2-cyano-3,12-dioxooleana-1,9(11)-dien-28-oate (bardoxolone methyl, CDDO-Me, (Fig. 3(b)) (Bore *et al.*, 2002), which is in phase 3 clinical trials for the treatment of chronic kidney disease in type 2 diabetic patients. It is essential to further study the role of ring strain in these drugs to understand their chemical reactivity as Michael acceptors because it is closely related to their biological potency (Zheng *et al.*, 2012). Ring A (C6A–C11A or C6B–C11B) assumes a slightly distorted envelope conformation (*Q* = 0.4295 (2) or 0.433 (2), θ = 124.8 (3)° or 57.7 (3)°, φ = 120.7 (3)° or 304.7 (3)° for the 1^st^ and 2^nd^ molecule, respectively) (Cremer & Pople, 1975). A typical envelope conformation in this case would maintain a θ value of 54.7° (or 180° - 54.7° = 125.3°) so the distortion is minor. Ring A maintains this conformation due to the rigid C*sp*^2^ hybridization at C7A, C8A, and C9A or C7B, C8B and C9B, respectively. Atoms C6A and C10A or C6B and C10B deviate from the Cremer-Pople plane by 0.204 (2) Å and 0.200 (2) Å or 0.215 (2) Å and 0.185 (2) Å, for the 1^st^ or 2^nd^ molecule, respectively. This deviation is most likely influenced by ring B. Ring B (C5A/C6A/C11A—C14A or C5B/C6B/C11B—C14B) is found to be in a slightly distorted cyclohexane chair conformation: *Q* = 0.5901 (2) or 0.5914 (2), θ = 6.93 (2)° or 173.74 (2)°. A typical θ value for a chair conformtion is 0.00° or 180°. The rigid dienone functionality in ring C (C1A—C5A/C14A or C1B—C5B/C14B) leads to a fully planar geometry (r.m.s.d. = 0.0111 (9) Å or 0.0167 (2) Å for the 1^st^ and 2^nd^ molecule). Therefore, in summary, while both rings A and B are distorted from ideal conformations, Ring C, has a very slight deviation from planarity.

Cremer-Pople analysis, therefore, supports the assignments of the ring systems in TBE-31 as envelope-chair-planar. The distortions from the ideal parameters can be attributed to the result of the rigidity of ring C. The X-ray structure reveals that the methyl group at C6 and hydrogen at C11 is *trans* and that the methyl group at C6 and alkyne group at C14 is *cis*. Consequently, two 1,3-diaxial interactions between the methyl group on C6 and ethynyl group on C14 and between the methyl groups on C6 and C10 are observed. In addition, the ethynyl group at C14 bends slightly off from the axial methyl group at C6 because of the 1,3-diaxial repulsion between both groups. These observations contribute to the higher strain seen in TBE-31 as compared to the all-*trans* perhydrophenanthrene with all chairs, (Marcos *et al.*, 2005). Overall, the X-ray structure of TBE-31 indicates that the unusually unsaturated tricyclic ring systems containing eight *sp*^2^ carbons and two 1,3-diaxial interactions, which are closely affected with each other, impose rigid constraints on the conformation of TBE-31. This high strain would increase the reactivity of the nonenolizable cyanoenone in ring C in comparison with that of MCE-1, because MCE-1, which is monocyclic, does not have such strain.

### Experimental

The title compound, C_21_H_18_N_2_O_2_, was synthesized in 14 steps from cyclohexanone, as described by Honda *et al.* (2007 and 2011). Recrystallization from methanol gave colorless rectangular crystals (m.p. 502–504 K).

### Refinement

All of the H atoms were positioned geometrically and then refined using the riding model with C—H lengths of 0.96 Å (CH), 0.97 Å (CH_2_) or 0.93 Å (CH_3_). The isotropic displacement parameters for these atoms were set to 1.20 (CH, CH_2_) or 1.50 (CH_3_) times *U*_eq_ of the parent atom. Weak high angle reflections (2*θ* > 140°) with intensity less than 2 *σ*(*I*) were omitted in the final refinement.

### Crystal data

**Table:**

C_21_H_18_N_2_O_2_	*Z* = 4
*M**_r_* = 330.37	*F*(000) = 696
Triclinic, *P*1	*D*_x_ = 1.270 Mg m^−^^3^
Hall symbol: -P 1	Cu *K*α radiation, λ = 1.54178 Å
*a* = 7.3012 (2) Å	Cell parameters from 16712 reflections
*b* = 12.9843 (3) Å	θ = 4.0–73.2°
*c* = 18.4254 (4) Å	µ = 0.66 mm^−^^1^
α = 95.051 (2)°	*T* = 299 K
β = 96.284 (2)°	Prism, colourless
γ = 92.338 (2)°	0.71 × 0.46 × 0.29 mm
*V* = 1727.26 (7) Å^3^	

### Data collection

**Table:**

Oxford Diffraction Xcalibur Atlas Gemini diffractometer	6478 independent reflections
Radiation source: fine-focus sealed tube	5160 reflections with *I* > 2σ(*I*)
Graphite monochromator	*R*_int_ = 0.035
ω scans	θ_max_ = 69.5°, θ_min_ = 4.0°
Absorption correction: gaussian (*CrysAlis RED*; Oxford Diffraction, 2010)	*h* = −8→7
*T*_min_ = 0.745, *T*_max_ = 0.897	*k* = −15→15
33944 measured reflections	*l* = −22→22

### Refinement

**Table:**

Refinement on *F*^2^	Secondary atom site location: difference Fourier map
Least-squares matrix: full	Hydrogen site location: inferred from neighbouring sites
*R*[*F*^2^ > 2σ(*F*^2^)] = 0.050	H-atom parameters constrained
*wR*(*F*^2^) = 0.140	*w* = 1/[σ^2^(*F*_o_^2^) + (0.0731*P*)^2^ + 0.4667*P*] where *P* = (*F*_o_^2^ + 2*F*_c_^2^)/3
*S* = 1.03	(Δ/σ)_max_ < 0.001
6478 reflections	Δρ_max_ = 0.22 e Å^−^^3^
458 parameters	Δρ_min_ = −0.18 e Å^−^^3^
0 restraints	Extinction correction: *SHELXL*, Fc^*^=kFc[1+0.001xFc^2^λ^3^/sin(2θ)]^-1/4^
Primary atom site location: structure-invariant direct methods	Extinction coefficient: 0.0024 (4)

### Special details

**Table:**

Geometry. All e.s.d.'s (except the e.s.d. in the dihedral angle between two l.s. planes) are estimated using the full covariance matrix. The cell e.s.d.'s are taken into account individually in the estimation of e.s.d.'s in distances, angles and torsion angles; correlations between e.s.d.'s in cell parameters are only used when they are defined by crystal symmetry. An approximate (isotropic) treatment of cell e.s.d.'s is used for estimating e.s.d.'s involving l.s. planes.
Refinement. Refinement of *F*^2^ against ALL reflections. The weighted *R*-factor *wR* and goodness of fit *S* are based on *F*^2^, conventional *R*-factors *R* are based on *F*, with *F* set to zero for negative *F*^2^. The threshold expression of *F*^2^ > σ(*F*^2^) is used only for calculating *R*-factors(gt) *etc*. and is not relevant to the choice of reflections for refinement. *R*-factors based on *F*^2^ are statistically about twice as large as those based on *F*, and *R*- factors based on ALL data will be even larger.

### Fractional atomic coordinates and isotropic or equivalent isotropic displacement parameters (Å^2^)

**Table:**

	*x*	*y*	*z*	*U*_iso_*/*U*_eq_	
O1A	0.7242 (2)	0.61192 (10)	0.49832 (8)	0.0625 (4)	
O2A	0.6374 (2)	0.18345 (11)	0.77597 (7)	0.0649 (4)	
N1A	0.6155 (3)	0.61182 (13)	0.31626 (10)	0.0667 (5)	
N2A	0.8755 (3)	0.41471 (15)	0.84087 (9)	0.0693 (5)	
C1A	0.6816 (3)	0.37153 (14)	0.38940 (10)	0.0486 (5)	
H1A	0.6463	0.3461	0.3409	0.058*	
C2A	0.6841 (3)	0.47338 (13)	0.40640 (9)	0.0441 (4)	
C3A	0.7309 (3)	0.51902 (13)	0.48269 (10)	0.0423 (4)	
C4A	0.7798 (3)	0.44778 (13)	0.53745 (9)	0.0414 (4)	
H4A	0.8082	0.4750	0.5860	0.050*	
C5A	0.7864 (2)	0.34548 (12)	0.52233 (9)	0.0370 (4)	
C6A	0.8231 (2)	0.27223 (12)	0.58271 (9)	0.0387 (4)	
C7A	0.8644 (3)	0.33248 (13)	0.65657 (9)	0.0419 (4)	
H7A	0.9394	0.3928	0.6605	0.050*	
C8A	0.7985 (3)	0.30344 (13)	0.71694 (9)	0.0407 (4)	
C9A	0.6819 (3)	0.20774 (13)	0.71829 (9)	0.0429 (4)	
C10A	0.6279 (3)	0.13976 (13)	0.64671 (9)	0.0413 (4)	
C11A	0.6413 (2)	0.20507 (12)	0.58059 (9)	0.0373 (4)	
H11A	0.5445	0.2549	0.5840	0.045*	
C12A	0.5947 (3)	0.14469 (13)	0.50516 (9)	0.0458 (4)	
H12A	0.6948	0.1006	0.4951	0.055*	
H12B	0.4841	0.1007	0.5052	0.055*	
C13A	0.5641 (3)	0.21787 (14)	0.44515 (9)	0.0472 (4)	
H13A	0.4560	0.2567	0.4526	0.057*	
H13B	0.5406	0.1775	0.3979	0.057*	
C14A	0.7328 (3)	0.29491 (13)	0.44422 (9)	0.0430 (4)	
C15A	0.6422 (3)	0.54746 (14)	0.35364 (10)	0.0505 (5)	
C16A	0.8435 (3)	0.36536 (14)	0.78652 (10)	0.0493 (5)	
C17A	0.8861 (3)	0.24025 (14)	0.41381 (10)	0.0516 (5)	
C18A	1.0007 (4)	0.20171 (19)	0.38268 (13)	0.0751 (7)	
H18A	1.0918	0.1711	0.3579	0.090*	
C19A	0.9995 (3)	0.21202 (15)	0.57255 (11)	0.0500 (5)	
H19A	1.0412	0.1835	0.6174	0.075*	
H19B	1.0943	0.2583	0.5599	0.075*	
H19C	0.9718	0.1570	0.5340	0.075*	
C20A	0.4273 (3)	0.10060 (18)	0.64789 (12)	0.0628 (6)	
H20A	0.3491	0.1583	0.6485	0.094*	
H20B	0.4175	0.0650	0.6909	0.094*	
H20C	0.3896	0.0540	0.6050	0.094*	
C21A	0.7504 (3)	0.04632 (14)	0.64789 (11)	0.0549 (5)	
H21A	0.7189	0.0041	0.6854	0.082*	
H21B	0.8776	0.0702	0.6579	0.082*	
H21C	0.7311	0.0063	0.6011	0.082*	
O1B	0.2331 (2)	0.59090 (9)	−0.04320 (7)	0.0543 (4)	
O2B	0.3443 (3)	0.18811 (12)	0.26554 (7)	0.0739 (5)	
N1B	0.4173 (3)	0.59329 (14)	−0.20451 (10)	0.0731 (6)	
N2B	0.0835 (4)	0.40614 (15)	0.29028 (11)	0.0835 (7)	
C1B	0.3689 (3)	0.35541 (13)	−0.12918 (9)	0.0441 (4)	
H1B	0.4221	0.3321	−0.1708	0.053*	
C2B	0.3422 (3)	0.45595 (13)	−0.11789 (9)	0.0429 (4)	
C3B	0.2610 (2)	0.49869 (13)	−0.05254 (9)	0.0406 (4)	
C4B	0.2208 (2)	0.42658 (13)	0.00024 (9)	0.0407 (4)	
H4B	0.1804	0.4531	0.0439	0.049*	
C5B	0.2381 (2)	0.32459 (12)	−0.01003 (8)	0.0365 (4)	
C6B	0.2005 (2)	0.25142 (13)	0.04851 (9)	0.0391 (4)	
C7B	0.1386 (3)	0.31054 (14)	0.11468 (10)	0.0457 (4)	
H7B	0.0566	0.3625	0.1072	0.055*	
C8B	0.1951 (3)	0.29232 (13)	0.18311 (9)	0.0457 (4)	
C9B	0.3180 (3)	0.20864 (14)	0.20232 (10)	0.0495 (5)	
C10B	0.3991 (3)	0.14548 (14)	0.14088 (10)	0.0488 (5)	
C11B	0.3891 (3)	0.20508 (12)	0.07121 (9)	0.0385 (4)	
H11B	0.4752	0.2654	0.0846	0.046*	
C12B	0.4612 (3)	0.14779 (13)	0.00498 (9)	0.0439 (4)	
H12C	0.5753	0.1163	0.0207	0.053*	
H12D	0.3720	0.0931	−0.0164	0.053*	
C13B	0.4954 (3)	0.22156 (13)	−0.05243 (9)	0.0427 (4)	
H13C	0.5381	0.1831	−0.0944	0.051*	
H13D	0.5916	0.2730	−0.0321	0.051*	
C14B	0.3177 (3)	0.27718 (12)	−0.07820 (9)	0.0394 (4)	
C15B	0.3863 (3)	0.53055 (14)	−0.16767 (10)	0.0518 (5)	
C16B	0.1314 (3)	0.35449 (15)	0.24348 (11)	0.0575 (5)	
C17B	0.1887 (3)	0.20143 (14)	−0.12493 (10)	0.0484 (5)	
C18B	0.1001 (4)	0.14161 (19)	−0.16700 (12)	0.0741 (7)	
H18B	0.0296	0.0940	−0.2005	0.089*	
C19B	0.0366 (3)	0.17316 (16)	0.01889 (11)	0.0563 (5)	
H19D	−0.0096	0.1422	0.0591	0.085*	
H19E	0.0782	0.1202	−0.0143	0.085*	
H19F	−0.0599	0.2088	−0.0065	0.085*	
C20B	0.6023 (4)	0.1310 (2)	0.16788 (13)	0.0761 (7)	
H20D	0.6711	0.1960	0.1705	0.114*	
H20E	0.6520	0.0816	0.1344	0.114*	
H20F	0.6107	0.1062	0.2157	0.114*	
C21B	0.2943 (4)	0.03946 (15)	0.13001 (12)	0.0746 (7)	
H21D	0.3250	0.0023	0.1721	0.112*	
H21E	0.3283	0.0009	0.0871	0.112*	
H21F	0.1640	0.0489	0.1241	0.112*	

### Atomic displacement parameters (Å^2^)

**Table:**

	*U*^11^	*U*^22^	*U*^33^	*U*^12^	*U*^13^	*U*^23^
O1A	0.0943 (12)	0.0354 (7)	0.0570 (8)	0.0079 (7)	0.0032 (8)	0.0036 (6)
O2A	0.0980 (12)	0.0608 (9)	0.0384 (7)	−0.0080 (8)	0.0199 (7)	0.0098 (6)
N1A	0.0991 (15)	0.0513 (10)	0.0524 (10)	0.0162 (10)	0.0058 (10)	0.0175 (8)
N2A	0.0989 (16)	0.0633 (11)	0.0424 (9)	0.0044 (10)	0.0005 (9)	−0.0046 (8)
C1A	0.0721 (14)	0.0424 (10)	0.0319 (8)	0.0071 (9)	0.0039 (8)	0.0069 (7)
C2A	0.0551 (12)	0.0403 (9)	0.0391 (9)	0.0071 (8)	0.0071 (8)	0.0116 (7)
C3A	0.0467 (11)	0.0369 (9)	0.0446 (9)	0.0035 (7)	0.0086 (8)	0.0052 (7)
C4A	0.0505 (11)	0.0387 (9)	0.0348 (8)	0.0022 (8)	0.0056 (7)	0.0025 (7)
C5A	0.0411 (10)	0.0381 (8)	0.0327 (8)	0.0025 (7)	0.0054 (7)	0.0058 (7)
C6A	0.0462 (10)	0.0366 (8)	0.0338 (8)	0.0025 (7)	0.0040 (7)	0.0062 (7)
C7A	0.0477 (11)	0.0390 (9)	0.0379 (9)	−0.0004 (7)	−0.0008 (7)	0.0062 (7)
C8A	0.0487 (11)	0.0400 (9)	0.0331 (8)	0.0047 (7)	0.0016 (7)	0.0035 (7)
C9A	0.0516 (11)	0.0417 (9)	0.0374 (9)	0.0079 (8)	0.0072 (8)	0.0094 (7)
C10A	0.0498 (11)	0.0360 (8)	0.0387 (9)	−0.0003 (7)	0.0057 (7)	0.0073 (7)
C11A	0.0453 (10)	0.0319 (8)	0.0347 (8)	0.0039 (7)	0.0019 (7)	0.0052 (6)
C12A	0.0596 (12)	0.0370 (9)	0.0392 (9)	−0.0023 (8)	0.0005 (8)	0.0023 (7)
C13A	0.0631 (12)	0.0409 (9)	0.0352 (9)	0.0010 (8)	−0.0023 (8)	0.0013 (7)
C14A	0.0623 (12)	0.0346 (8)	0.0326 (8)	0.0073 (8)	0.0048 (8)	0.0043 (7)
C15A	0.0669 (13)	0.0424 (10)	0.0436 (10)	0.0095 (9)	0.0064 (9)	0.0086 (8)
C16A	0.0647 (13)	0.0444 (10)	0.0390 (10)	0.0049 (9)	0.0043 (9)	0.0070 (8)
C17A	0.0762 (15)	0.0426 (10)	0.0383 (9)	0.0085 (9)	0.0123 (9)	0.0061 (8)
C18A	0.102 (2)	0.0725 (15)	0.0586 (13)	0.0294 (14)	0.0306 (13)	0.0106 (11)
C19A	0.0501 (12)	0.0538 (11)	0.0492 (10)	0.0108 (9)	0.0073 (9)	0.0163 (9)
C20A	0.0626 (14)	0.0693 (14)	0.0571 (12)	−0.0130 (11)	0.0059 (10)	0.0174 (10)
C21A	0.0788 (15)	0.0401 (10)	0.0486 (11)	0.0117 (9)	0.0098 (10)	0.0128 (8)
O1B	0.0689 (9)	0.0358 (7)	0.0592 (8)	0.0092 (6)	0.0076 (7)	0.0065 (6)
O2B	0.1148 (14)	0.0751 (10)	0.0388 (8)	0.0313 (9)	0.0169 (8)	0.0212 (7)
N1B	0.1133 (17)	0.0531 (10)	0.0526 (10)	−0.0171 (10)	0.0080 (10)	0.0151 (8)
N2B	0.143 (2)	0.0590 (11)	0.0570 (11)	0.0193 (12)	0.0420 (12)	0.0056 (9)
C1B	0.0576 (12)	0.0432 (9)	0.0328 (8)	0.0024 (8)	0.0093 (8)	0.0053 (7)
C2B	0.0532 (11)	0.0392 (9)	0.0362 (9)	−0.0013 (8)	0.0029 (8)	0.0080 (7)
C3B	0.0422 (10)	0.0377 (9)	0.0406 (9)	0.0037 (7)	−0.0014 (7)	0.0037 (7)
C4B	0.0471 (11)	0.0405 (9)	0.0351 (8)	0.0075 (8)	0.0064 (7)	0.0028 (7)
C5B	0.0388 (9)	0.0399 (9)	0.0309 (8)	0.0031 (7)	0.0022 (7)	0.0056 (6)
C6B	0.0462 (10)	0.0375 (9)	0.0349 (8)	0.0040 (7)	0.0071 (7)	0.0062 (7)
C7B	0.0553 (12)	0.0436 (9)	0.0428 (10)	0.0114 (8)	0.0161 (8)	0.0127 (8)
C8B	0.0644 (13)	0.0384 (9)	0.0374 (9)	0.0053 (8)	0.0168 (8)	0.0066 (7)
C9B	0.0699 (13)	0.0432 (10)	0.0377 (9)	0.0058 (9)	0.0099 (9)	0.0105 (8)
C10B	0.0707 (13)	0.0396 (9)	0.0397 (9)	0.0147 (9)	0.0116 (9)	0.0120 (7)
C11B	0.0490 (11)	0.0337 (8)	0.0345 (8)	0.0056 (7)	0.0075 (7)	0.0068 (6)
C12B	0.0538 (11)	0.0386 (9)	0.0411 (9)	0.0116 (8)	0.0088 (8)	0.0056 (7)
C13B	0.0517 (11)	0.0411 (9)	0.0367 (9)	0.0064 (8)	0.0108 (8)	0.0028 (7)
C14B	0.0518 (11)	0.0340 (8)	0.0330 (8)	0.0033 (7)	0.0076 (7)	0.0032 (6)
C15B	0.0717 (14)	0.0416 (10)	0.0416 (10)	−0.0046 (9)	0.0055 (9)	0.0061 (8)
C16B	0.0911 (17)	0.0443 (10)	0.0419 (10)	0.0093 (10)	0.0219 (10)	0.0101 (8)
C17B	0.0637 (13)	0.0441 (10)	0.0374 (9)	0.0017 (9)	0.0035 (9)	0.0066 (8)
C18B	0.099 (2)	0.0655 (14)	0.0514 (12)	−0.0145 (13)	−0.0106 (12)	0.0025 (11)
C19B	0.0561 (13)	0.0623 (12)	0.0515 (11)	−0.0109 (10)	0.0088 (9)	0.0132 (9)
C20B	0.0853 (18)	0.0932 (18)	0.0565 (13)	0.0401 (14)	0.0086 (12)	0.0275 (12)
C21B	0.133 (2)	0.0385 (11)	0.0569 (13)	0.0057 (12)	0.0219 (14)	0.0154 (9)

### Geometric parameters (Å, º)

**Table:**

O1A—C3A	1.219 (2)	O1B—C3B	1.223 (2)
O2A—C9A	1.208 (2)	O2B—C9B	1.214 (2)
N1A—C15A	1.138 (2)	N1B—C15B	1.136 (2)
N2A—C16A	1.137 (2)	N2B—C16B	1.139 (3)
C1A—C2A	1.331 (2)	C1B—C2B	1.329 (2)
C1A—C14A	1.508 (2)	C1B—C14B	1.506 (2)
C1A—H1A	0.9300	C1B—H1B	0.9300
C2A—C15A	1.445 (2)	C2B—C15B	1.442 (2)
C2A—C3A	1.475 (2)	C2B—C3B	1.475 (2)
C3A—C4A	1.454 (2)	C3B—C4B	1.452 (2)
C4A—C5A	1.337 (2)	C4B—C5B	1.334 (2)
C4A—H4A	0.9300	C4B—H4B	0.9300
C5A—C14A	1.531 (2)	C5B—C14B	1.532 (2)
C5A—C6A	1.535 (2)	C5B—C6B	1.538 (2)
C6A—C7A	1.503 (2)	C6B—C7B	1.505 (2)
C6A—C11A	1.553 (2)	C6B—C11B	1.555 (2)
C6A—C19A	1.553 (3)	C6B—C19B	1.556 (3)
C7A—C8A	1.337 (2)	C7B—C8B	1.327 (3)
C7A—H7A	0.9300	C7B—H7B	0.9300
C8A—C16A	1.450 (2)	C8B—C16B	1.446 (3)
C8A—C9A	1.481 (3)	C8B—C9B	1.480 (3)
C9A—C10A	1.525 (2)	C9B—C10B	1.526 (3)
C10A—C20A	1.534 (3)	C10B—C21B	1.534 (3)
C10A—C21A	1.536 (3)	C10B—C20B	1.537 (3)
C10A—C11A	1.554 (2)	C10B—C11B	1.553 (2)
C11A—C12A	1.531 (2)	C11B—C12B	1.527 (2)
C11A—H11A	0.9800	C11B—H11B	0.9800
C12A—C13A	1.524 (2)	C12B—C13B	1.522 (2)
C12A—H12A	0.9700	C12B—H12C	0.9700
C12A—H12B	0.9700	C12B—H12D	0.9700
C13A—C14A	1.557 (3)	C13B—C14B	1.559 (3)
C13A—H13A	0.9700	C13B—H13C	0.9700
C13A—H13B	0.9700	C13B—H13D	0.9700
C14A—C17A	1.484 (3)	C14B—C17B	1.481 (3)
C17A—C18A	1.170 (3)	C17B—C18B	1.169 (3)
C18A—H18A	0.9300	C18B—H18B	0.9300
C19A—H19A	0.9600	C19B—H19D	0.9600
C19A—H19B	0.9600	C19B—H19E	0.9600
C19A—H19C	0.9600	C19B—H19F	0.9600
C20A—H20A	0.9600	C20B—H20D	0.9600
C20A—H20B	0.9600	C20B—H20E	0.9600
C20A—H20C	0.9600	C20B—H20F	0.9600
C21A—H21A	0.9600	C21B—H21D	0.9600
C21A—H21B	0.9600	C21B—H21E	0.9600
C21A—H21C	0.9600	C21B—H21F	0.9600
			
C2A—C1A—C14A	123.82 (16)	C2B—C1B—C14B	123.56 (16)
C2A—C1A—H1A	118.1	C2B—C1B—H1B	118.2
C14A—C1A—H1A	118.1	C14B—C1B—H1B	118.2
C1A—C2A—C15A	124.07 (17)	C1B—C2B—C15B	123.65 (17)
C1A—C2A—C3A	121.19 (16)	C1B—C2B—C3B	121.25 (16)
C15A—C2A—C3A	114.74 (15)	C15B—C2B—C3B	115.09 (15)
O1A—C3A—C4A	122.47 (16)	O1B—C3B—C4B	122.28 (16)
O1A—C3A—C2A	120.75 (16)	O1B—C3B—C2B	120.98 (16)
C4A—C3A—C2A	116.76 (15)	C4B—C3B—C2B	116.73 (14)
C5A—C4A—C3A	124.00 (16)	C5B—C4B—C3B	124.08 (15)
C5A—C4A—H4A	118.0	C5B—C4B—H4B	118.0
C3A—C4A—H4A	118.0	C3B—C4B—H4B	118.0
C4A—C5A—C14A	120.71 (15)	C4B—C5B—C14B	120.39 (15)
C4A—C5A—C6A	122.14 (15)	C4B—C5B—C6B	122.36 (14)
C14A—C5A—C6A	116.69 (13)	C14B—C5B—C6B	116.92 (13)
C7A—C6A—C5A	110.68 (13)	C7B—C6B—C5B	110.75 (13)
C7A—C6A—C11A	109.43 (14)	C7B—C6B—C11B	108.83 (14)
C5A—C6A—C11A	105.41 (13)	C5B—C6B—C11B	105.36 (13)
C7A—C6A—C19A	104.61 (14)	C7B—C6B—C19B	105.03 (15)
C5A—C6A—C19A	110.83 (14)	C5B—C6B—C19B	110.11 (14)
C11A—C6A—C19A	115.95 (14)	C11B—C6B—C19B	116.80 (15)
C8A—C7A—C6A	123.10 (16)	C8B—C7B—C6B	123.41 (16)
C8A—C7A—H7A	118.5	C8B—C7B—H7B	118.3
C6A—C7A—H7A	118.5	C6B—C7B—H7B	118.3
C7A—C8A—C16A	120.27 (17)	C7B—C8B—C16B	119.65 (18)
C7A—C8A—C9A	123.61 (16)	C7B—C8B—C9B	123.64 (16)
C16A—C8A—C9A	116.11 (15)	C16B—C8B—C9B	116.69 (16)
O2A—C9A—C8A	119.60 (16)	O2B—C9B—C8B	119.72 (17)
O2A—C9A—C10A	121.65 (16)	O2B—C9B—C10B	121.47 (17)
C8A—C9A—C10A	118.69 (14)	C8B—C9B—C10B	118.69 (15)
C9A—C10A—C20A	106.60 (15)	C9B—C10B—C21B	106.79 (17)
C9A—C10A—C21A	107.22 (15)	C9B—C10B—C20B	107.19 (17)
C20A—C10A—C21A	108.35 (16)	C21B—C10B—C20B	109.00 (19)
C9A—C10A—C11A	109.79 (13)	C9B—C10B—C11B	110.05 (14)
C20A—C10A—C11A	109.59 (15)	C21B—C10B—C11B	114.39 (16)
C21A—C10A—C11A	114.95 (15)	C20B—C10B—C11B	109.17 (17)
C12A—C11A—C6A	110.22 (14)	C12B—C11B—C10B	114.85 (13)
C12A—C11A—C10A	114.83 (13)	C12B—C11B—C6B	110.69 (14)
C6A—C11A—C10A	115.50 (14)	C10B—C11B—C6B	115.74 (14)
C12A—C11A—H11A	105.0	C12B—C11B—H11B	104.7
C6A—C11A—H11A	105.0	C10B—C11B—H11B	104.7
C10A—C11A—H11A	105.0	C6B—C11B—H11B	104.7
C13A—C12A—C11A	111.05 (14)	C13B—C12B—C11B	110.78 (13)
C13A—C12A—H12A	109.4	C13B—C12B—H12C	109.5
C11A—C12A—H12A	109.4	C11B—C12B—H12C	109.5
C13A—C12A—H12B	109.4	C13B—C12B—H12D	109.5
C11A—C12A—H12B	109.4	C11B—C12B—H12D	109.5
H12A—C12A—H12B	108.0	H12C—C12B—H12D	108.1
C12A—C13A—C14A	112.38 (15)	C12B—C13B—C14B	111.87 (15)
C12A—C13A—H13A	109.1	C12B—C13B—H13C	109.2
C14A—C13A—H13A	109.1	C14B—C13B—H13C	109.2
C12A—C13A—H13B	109.1	C12B—C13B—H13D	109.2
C14A—C13A—H13B	109.1	C14B—C13B—H13D	109.2
H13A—C13A—H13B	107.9	H13C—C13B—H13D	107.9
C17A—C14A—C1A	103.64 (15)	C17B—C14B—C1B	104.38 (14)
C17A—C14A—C5A	112.85 (16)	C17B—C14B—C5B	113.64 (15)
C1A—C14A—C5A	113.42 (14)	C1B—C14B—C5B	113.76 (13)
C17A—C14A—C13A	110.09 (15)	C17B—C14B—C13B	108.64 (14)
C1A—C14A—C13A	108.47 (16)	C1B—C14B—C13B	108.01 (15)
C5A—C14A—C13A	108.25 (14)	C5B—C14B—C13B	108.18 (13)
N1A—C15A—C2A	174.5 (2)	N1B—C15B—C2B	176.3 (2)
N2A—C16A—C8A	178.7 (2)	N2B—C16B—C8B	177.9 (2)
C18A—C17A—C14A	172.5 (2)	C18B—C17B—C14B	173.0 (2)
C17A—C18A—H18A	180.0	C17B—C18B—H18B	180.0
C6A—C19A—H19A	109.5	C6B—C19B—H19D	109.5
C6A—C19A—H19B	109.5	C6B—C19B—H19E	109.5
H19A—C19A—H19B	109.5	H19D—C19B—H19E	109.5
C6A—C19A—H19C	109.5	C6B—C19B—H19F	109.5
H19A—C19A—H19C	109.5	H19D—C19B—H19F	109.5
H19B—C19A—H19C	109.5	H19E—C19B—H19F	109.5
C10A—C20A—H20A	109.5	C10B—C20B—H20D	109.5
C10A—C20A—H20B	109.5	C10B—C20B—H20E	109.5
H20A—C20A—H20B	109.5	H20D—C20B—H20E	109.5
C10A—C20A—H20C	109.5	C10B—C20B—H20F	109.5
H20A—C20A—H20C	109.5	H20D—C20B—H20F	109.5
H20B—C20A—H20C	109.5	H20E—C20B—H20F	109.5
C10A—C21A—H21A	109.5	C10B—C21B—H21D	109.5
C10A—C21A—H21B	109.5	C10B—C21B—H21E	109.5
H21A—C21A—H21B	109.5	H21D—C21B—H21E	109.5
C10A—C21A—H21C	109.5	C10B—C21B—H21F	109.5
H21A—C21A—H21C	109.5	H21D—C21B—H21F	109.5
H21B—C21A—H21C	109.5	H21E—C21B—H21F	109.5
			
C14A—C1A—C2A—C15A	177.48 (19)	C14B—C1B—C2B—C15B	−178.20 (18)
C14A—C1A—C2A—C3A	−2.6 (3)	C14B—C1B—C2B—C3B	0.9 (3)
C1A—C2A—C3A—O1A	−176.5 (2)	C1B—C2B—C3B—O1B	−178.44 (18)
C15A—C2A—C3A—O1A	3.4 (3)	C15B—C2B—C3B—O1B	0.7 (3)
C1A—C2A—C3A—C4A	2.0 (3)	C1B—C2B—C3B—C4B	3.0 (3)
C15A—C2A—C3A—C4A	−178.11 (17)	C15B—C2B—C3B—C4B	−177.85 (16)
O1A—C3A—C4A—C5A	179.23 (19)	O1B—C3B—C4B—C5B	175.56 (18)
C2A—C3A—C4A—C5A	0.8 (3)	C2B—C3B—C4B—C5B	−5.9 (3)
C3A—C4A—C5A—C14A	−2.8 (3)	C3B—C4B—C5B—C14B	4.6 (3)
C3A—C4A—C5A—C6A	−174.82 (16)	C3B—C4B—C5B—C6B	177.81 (16)
C4A—C5A—C6A—C7A	−4.8 (2)	C4B—C5B—C6B—C7B	1.7 (2)
C14A—C5A—C6A—C7A	−177.07 (15)	C14B—C5B—C6B—C7B	175.09 (15)
C4A—C5A—C6A—C11A	113.46 (18)	C4B—C5B—C6B—C11B	−115.85 (18)
C14A—C5A—C6A—C11A	−58.85 (19)	C14B—C5B—C6B—C11B	57.58 (18)
C4A—C5A—C6A—C19A	−120.35 (19)	C4B—C5B—C6B—C19B	117.39 (19)
C14A—C5A—C6A—C19A	67.3 (2)	C14B—C5B—C6B—C19B	−69.2 (2)
C5A—C6A—C7A—C8A	138.96 (18)	C5B—C6B—C7B—C8B	−139.05 (19)
C11A—C6A—C7A—C8A	23.2 (2)	C11B—C6B—C7B—C8B	−23.7 (2)
C19A—C6A—C7A—C8A	−101.6 (2)	C19B—C6B—C7B—C8B	102.1 (2)
C6A—C7A—C8A—C16A	−179.86 (16)	C6B—C7B—C8B—C16B	178.71 (18)
C6A—C7A—C8A—C9A	1.6 (3)	C6B—C7B—C8B—C9B	−3.1 (3)
C7A—C8A—C9A—O2A	175.28 (18)	C7B—C8B—C9B—O2B	−170.4 (2)
C16A—C8A—C9A—O2A	−3.3 (3)	C16B—C8B—C9B—O2B	7.8 (3)
C7A—C8A—C9A—C10A	−2.1 (3)	C7B—C8B—C9B—C10B	5.7 (3)
C16A—C8A—C9A—C10A	179.36 (16)	C16B—C8B—C9B—C10B	−176.02 (18)
O2A—C9A—C10A—C20A	41.6 (2)	O2B—C9B—C10B—C21B	70.5 (3)
C8A—C9A—C10A—C20A	−141.06 (17)	C8B—C9B—C10B—C21B	−105.6 (2)
O2A—C9A—C10A—C21A	−74.2 (2)	O2B—C9B—C10B—C20B	−46.2 (3)
C8A—C9A—C10A—C21A	103.07 (18)	C8B—C9B—C10B—C20B	137.69 (19)
O2A—C9A—C10A—C11A	160.26 (18)	O2B—C9B—C10B—C11B	−164.8 (2)
C8A—C9A—C10A—C11A	−22.4 (2)	C8B—C9B—C10B—C11B	19.1 (3)
C7A—C6A—C11A—C12A	178.85 (13)	C9B—C10B—C11B—C12B	−178.19 (17)
C5A—C6A—C11A—C12A	59.80 (17)	C21B—C10B—C11B—C12B	−58.0 (2)
C19A—C6A—C11A—C12A	−63.18 (18)	C20B—C10B—C11B—C12B	64.4 (2)
C7A—C6A—C11A—C10A	−48.96 (18)	C9B—C10B—C11B—C6B	−47.2 (2)
C5A—C6A—C11A—C10A	−168.01 (13)	C21B—C10B—C11B—C6B	73.0 (2)
C19A—C6A—C11A—C10A	69.01 (19)	C20B—C10B—C11B—C6B	−164.62 (16)
C9A—C10A—C11A—C12A	178.62 (15)	C7B—C6B—C11B—C12B	−177.78 (14)
C20A—C10A—C11A—C12A	−64.6 (2)	C5B—C6B—C11B—C12B	−58.97 (17)
C21A—C10A—C11A—C12A	57.7 (2)	C19B—C6B—C11B—C12B	63.59 (19)
C9A—C10A—C11A—C6A	48.63 (19)	C7B—C6B—C11B—C10B	49.32 (19)
C20A—C10A—C11A—C6A	165.39 (15)	C5B—C6B—C11B—C10B	168.12 (14)
C21A—C10A—C11A—C6A	−72.33 (19)	C19B—C6B—C11B—C10B	−69.31 (19)
C6A—C11A—C12A—C13A	−61.7 (2)	C10B—C11B—C12B—C13B	−164.38 (16)
C10A—C11A—C12A—C13A	165.77 (16)	C6B—C11B—C12B—C13B	62.27 (19)
C11A—C12A—C13A—C14A	56.2 (2)	C11B—C12B—C13B—C14B	−57.5 (2)
C2A—C1A—C14A—C17A	−122.1 (2)	C2B—C1B—C14B—C17B	122.2 (2)
C2A—C1A—C14A—C5A	0.6 (3)	C2B—C1B—C14B—C5B	−2.2 (3)
C2A—C1A—C14A—C13A	120.9 (2)	C2B—C1B—C14B—C13B	−122.32 (19)
C4A—C5A—C14A—C17A	119.57 (18)	C4B—C5B—C14B—C17B	−119.76 (18)
C6A—C5A—C14A—C17A	−68.0 (2)	C6B—C5B—C14B—C17B	66.67 (19)
C4A—C5A—C14A—C1A	2.1 (3)	C4B—C5B—C14B—C1B	−0.5 (2)
C6A—C5A—C14A—C1A	174.52 (16)	C6B—C5B—C14B—C1B	−174.06 (15)
C4A—C5A—C14A—C13A	−118.33 (18)	C4B—C5B—C14B—C13B	119.51 (17)
C6A—C5A—C14A—C13A	54.1 (2)	C6B—C5B—C14B—C13B	−54.06 (19)
C12A—C13A—C14A—C17A	73.72 (18)	C12B—C13B—C14B—C17B	−72.61 (17)
C12A—C13A—C14A—C1A	−173.52 (15)	C12B—C13B—C14B—C1B	174.72 (14)
C12A—C13A—C14A—C5A	−50.06 (19)	C12B—C13B—C14B—C5B	51.18 (18)

### Hydrogen-bond geometry (Å, º)

**Table:**

*D*—H···*A*	*D*—H	H···*A*	*D*···*A*	*D*—H···*A*
C4*A*—H4*A*···N2*B*^i^	0.93	2.66	3.572 (3)	166
C4*B*—H4*B*···N2*A*^i^	0.93	2.69	3.580 (2)	161
C7*B*—H7*B*···O1*B*^ii^	0.93	2.43	3.246 (2)	146
C13*B*—H13*D*···O1*B*^iii^	0.97	2.38	3.324 (2)	163
C13*B*—H13*C*···O2*A*^iv^	0.97	2.57	3.435 (2)	148
C13*A*—H13*A*···O1*A*^i^	0.97	2.37	3.295 (2)	159

Symmetry codes: (i) −*x*+1, −*y*+1, −*z*+1; (ii) −*x*, −*y*+1, −*z*; (iii) −*x*+1, −*y*+1, −*z*; (iv) *x*, *y*, *z*−1.
